# A new species of *Fordiophyton* (Sonerileae, Melastomataceae) from Yunnan, China

**DOI:** 10.3897/phytokeys.122.35260

**Published:** 2019-05-28

**Authors:** Jin-Hong Dai, Qiu-Jie Zhou, Zhi-Yong Yu, Ren-Chao Zhou, Ying Liu

**Affiliations:** 1 State Key Laboratory of Biocontrol and Guangdong Key Laboratory of Plant Resources, School of Life Sciences, Sun Yat-sen University, No. 135, Xin-Gang-Xi Road, Guangzhou 510275, China Sun Yat-sen University Guangzhou China; 2 Management Bureau of Fenshuiling National Nature Reserve, Jinping 661500, China Management Bureau of Fenshuiling National Nature Reserve Jinping China

**Keywords:** *
Fordiophyton
*, Melastomataceae, taxonomy, phylogeny

## Abstract

*Fordiophytonjinpingense* (Melastomataceae; Sonerileae), a species occurring in south-eastern Yunnan, China, is described as new, based on morphological and molecular data. Phylogenetic analyses, based on nrITS sequence data, showed that, except *F.breviscapum*, all species sampled in *Fordiophyton* formed a strongly supported clade in which two geographical lineages were recovered. The generic placement of *F.jinpingense* is well supported by phylogenetic analyses and a character combination of 4-merous flowers, distinctly dimorphic stamens and the connectives basally not calcarate. Molecular divergence and morphological evidence indicate that *F.jinpingense* is well separated from other members of the genus, thus justifying its recognition as a distinct species. *Fordiophytonjinpingense* is phylogenetically closest to *F.repens*, but differs markedly from the latter in stem morphology (short, obtusely 4-sided vs. long, 4-angular), habit (erect vs. creeping), leaf size (6–16.5 × 4.5–13 cm vs. 4–7.5 × 4–6.5 cm) and flower number per inflorescence (5–13 vs. 3–6).

## Introduction

In the study of Asian Sonerileae, Stapf established two new genera, *Fordiophyton* Stapf and *Gymnagathis* Stapf, based on three species in China (Stapf 1892). Both genera were accepted by subsequent authors (Krasser 1893; Diels 1932; Li 1944; Chen 1984a, b). Li (1944) pointed out that *Gymnagathis* is an illegitimate generic name and proposed a new name *Stapfiophyton* Li to replace it. However, Hansen (1992) considered the type species of *Stapfiophyton*, *S.peperomiifolium* (Oliver) H. L. Li, to be similar to *Fordiophyton* and therefore placed *Stapfiophyton* in synonymy under *Fordiophyton*. Hansen’s treatment was thereafter adopted by other authors (Deng and Wu 2004; Chen and Renner 2007).

**Figure 1.**
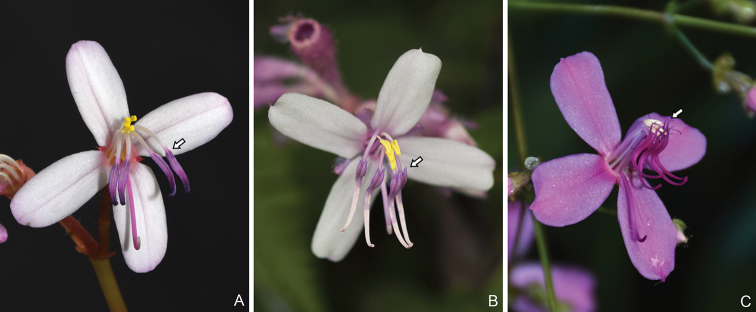
Flowers of *Fordiophyton* showing anther morphology of the longer stamens. **A***F.peperomiifolium* from Qingyuan, Guangdong, China, anther base not forked **B***F.faberi* from Hengshan, Hunan, China, anther base forked **C***F.strictum* from Pingbian, Yunnan, China, anther base forked and curved. Arrows indicate the anther base of the longer stamens.

*Fordiophyton*, as currently defined, is a small Asian genus of 13 species mainly occurring in southern China, with only one species extending to northern Vietnam (Chen and Renner 2007; Ning and Liu 2010; Zeng et al. 2016a, b). It is characterised by 4-merous flowers, eight unequal stamens, distinctly dimorphic anthers, connectives not calcarate at the base and anther base of longer stamens not forked, obtusely forked or forked and curved (Fig. 1). Ten species of *Fordiophyton* have been included in previous molecular phylogenetic studies (Zeng et al. 2016a, b; Zhou et al. in press). Amongst the species sampled in *Fordiophyton*, *F.breviscapum* (C. Chen) Y. F. Deng & T. L. Wu appeared to be close to *Phyllagathistetrandra* Diels and *P.elattandra* Diels (Zhou et al. in press), while the remaining species, including the type species, *F.faberi* Stapf, formed a well-supported clade close to *Blastus*, *Bredia*-*Phyllagathis* clade 2 and *Plagiopetalum* (Zeng et al. 2016a, b; Zhou et al. in press).

**Figure 2.**
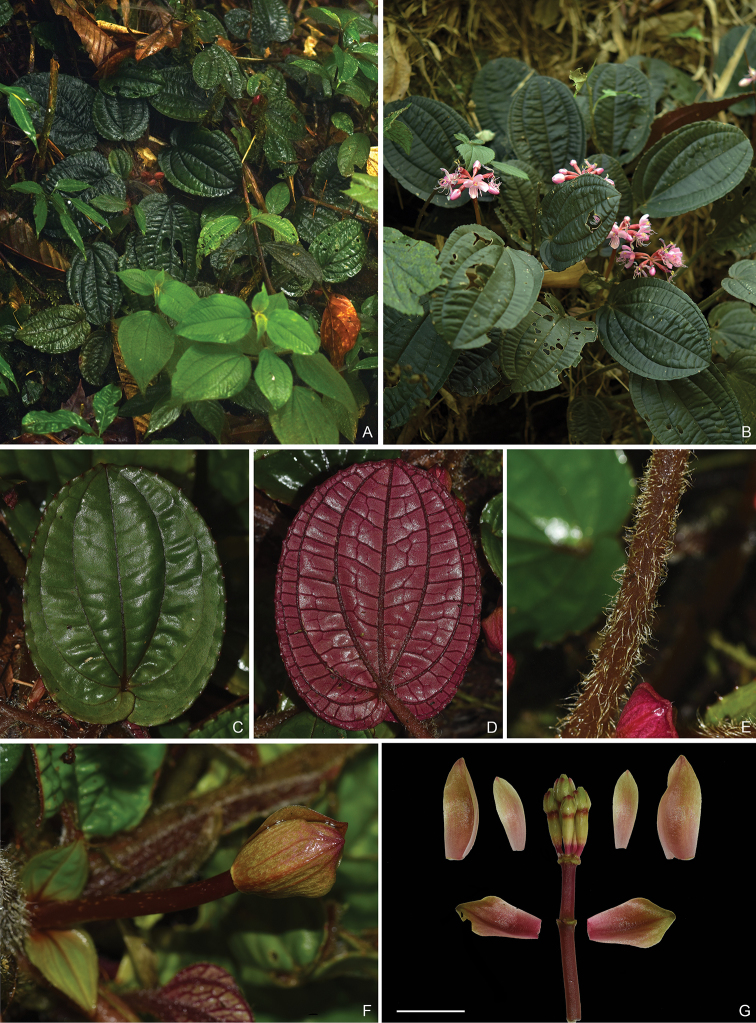
*Fordiophytonjinpingense*, all from Y. Liu 728 (SYS, A). **A** Habitat **B** a flowering individual **C** adaxial leaf surface **D** abaxial leaf surface **E** petiole villous with multiseriate hairs **F** young inflorescence **G** young inflorescence dissected showing the position and morphology of bracts. Scale bar: 2 cm (**G**).

**Figure 3.**
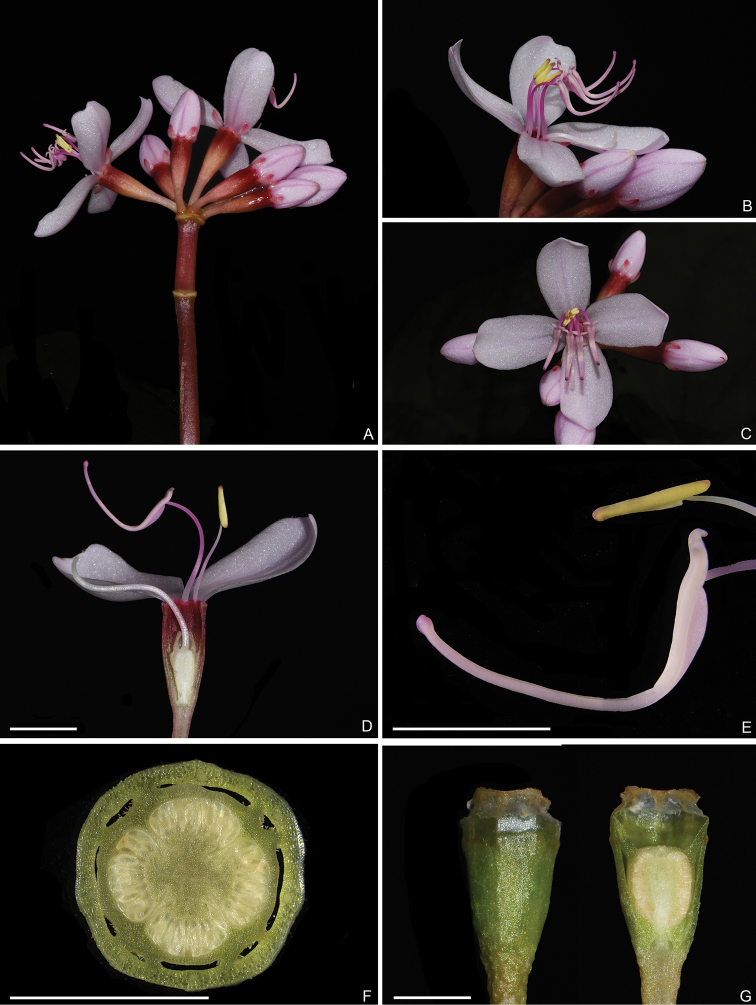
Detail of inflorescence, flower, stamens, ovary and fruit of *Fordiophytonjinpingense*, all from Y. Liu 728 (SYS, A). **A** Mature inflorescence **B** side view of a flower **C** top view of a flower **D** longitudinal section of a flower showing dimorphic stamens and ovary crown **E** anther morphology in detail **F** transection of ovary at young fruit stage, showing the very short-stalked, nearly sessile placenta **G** young fruit showing the crown not exserted from hypanthium. Scale bars: 5 mm (**D–F**).

During a field survey, we encountered a distinct plant in the forests of Ma-an-di, Fenshuiling National Nature Reserve in Jinping County, south-eastern Yunnan. This plant had eight distinctly dimorphic stamens and connectives not calcarate at the base, which are typical characteristics of *Fordiophyton*. It was distinct from all known species of *Fordiophyton* in the combination of short stems with distinct internodes, basal rosette of leaves, unwinged, densely villous petioles, umbellate inflorescence and anther base of longer stamens distinctly forked and curved (Figs 2, 3). We suspected that it represented an undescribed species.

To evaluate the specific status and phylogenetic position of this species in *Fordiophyton*, phylogenetic analyses were performed, based on DNA sequence data of the nuclear ribosomal internal transcribed spacer (nrITS). The results confirmed our suspicions that these plants represented a previously unrecognised species, *F.jinpingense*, which we describe below as new. A key to separate it from other species of *Fordiophyton* is also provided.

## Materials and methods

For phylogenetic analyses, the nrITS sequences of *F.longipes* and *F.jinpingense* were newly sequenced, while the sequences of other species were downloaded from GenBank. The final dataset contained 131 accessions representing 106 species and three varieties from 19 genera in Sonerileae/Dissochaeteae and one in tribe Blakeeae. *Blakeaschlimii* (Naudin) Triana was selected as an outgroup according to previous studies (Clausing et al. 2000; Clausing and Renner 2001; Renner et al. 2001; Goldenberg et al. 2012; Zhou et al. in press). In total, twelve species of *Fordiophyton* (85.7%) were sampled in the analyses. The source of the materials and GenBank accession numbers are given in Suppl. material 1.

Total DNA was extracted from fresh leaves using the modified CTAB procedure (Doyle and Doyle 1987). The nrITS region of *F.longipes* and *F.jinpingense* were amplified and sequenced using universal primers (White et al. 1990), following the procedure described in Zou et al. (2017).

Sequences were aligned using SeqMan v.7.1.0 (DNASTAR Inc., Madison, WI). The best-fitting nucleotide substitution model was determined using the Akaike Information Criterion in Modeltest version 3.7 (Posada and Crandall 1998) prior to phylogenetic analyses. The substitution model GTR+I+G was selected. Bayesian Inference (BI), Maximum Likelihood (ML) and Maximum Parsimony (MP) analyses were performed according to Zhou et al. (in press).

## Results

**Figure 4.**
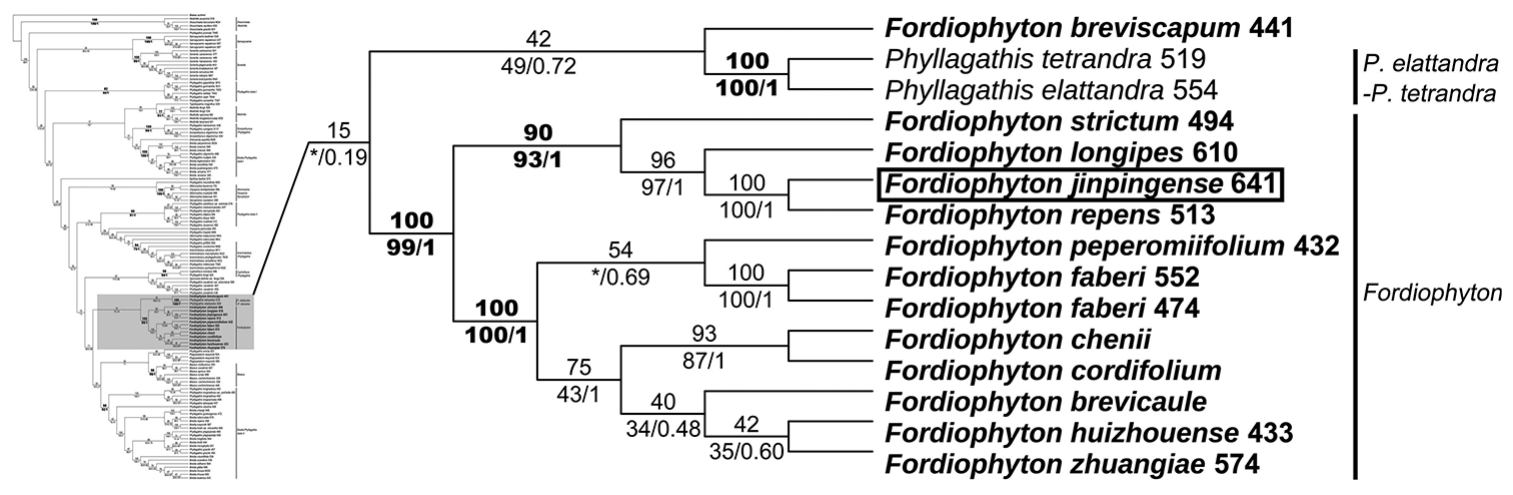
Phylogenetic relationships amongst species of *Fordiophyton*. Part of the Maximum Likelihood (ML) phylogenetic tree based on nrITS sequence data. Numbers above branches are bootstrap values obtained from maximum likelihood analyses, and those below branches are Bayesian posterior probabilities (right) and bootstrap values (left) resulting from maximum parsimony analyses. Box denotes the new species; asterisk denotes a branch collapsed in Bayesian inference or maximum parsimony analyses.

The aligned sequence matrix contained 766 characters. Statistics of sequences sampled are summarised in Suppl. material 2. Trees generated by ML, MP and BI analyses were highly similar in topology, except that some nodes with weak support in ML analyses collapsed in MP or BI analyses. The tree resulting from ML analysis is shown in Suppl. material 3, with BI posterior probability (PP), ML bootstrap support values (BS) and MP bootstrap support values (PBS) labelled at nodes. As shown in Fig. 4, *F.breviscapum*, *P.tetrandra* and *P.elattandra* comprised a clade with weak support (PP = 0.72, BS = 42%, PBS = 49%), while the remaining 11 species formed the well-supported *Fordiophyton* clade (PP = 1.0, BS = 100%, PBS = 99%). The sister relationship of these two clades was only weakly supported in BI and ML analyses (PP = 0.19, BS= 15%). Two subgroups were recovered within the *Fordiophyton* clade with strong support. One subgroup included seven species, namely *F.brevicaule* C. Chen, *F.chenii* S. Jin Zeng & X. Y. Zhuang, *F.cordifolium* C. Y. Wu ex C. Chen, *F.faberi*, *F.huizhouense* S. Jin Zeng & X. Y. Zhuang, *F.peperomiifolium* (Oliv.) C. Hansen and *F.zhuangiae* S. Jin Zeng & G. D. Tang (PP = 1.0, BS = 100%, PBS = 100%); the other contained *F.longipes* Y. C. Huang, *F.repens* Y. C. Huang, *F.strictum* Diels and the new species, *F.jinpingense* (PP = 1.0, BS = 90%, PBS = 93%) (Fig. 4).

## Discussion

### Phylogeny of *Fordiophyton*

Phylogenetic analyses recovered two subclades in the *Fordiophyton* clade (Fig. 4). The grouping of species shows weak correlation with morphology. Both subclades are quite variable in habit (short stem with a basal rosette of leaves/long and leafy stem) and morphology of the leaf blade (ovate, cordate to lanceolate), petiole (hairy/glabrous, winged/unwinged) and inflorescence (umbellate/cymose paniculate). However, the subclades represent two geographic lineages. Six out of the seven species in subclade 1 are narrowly endemic to south-eastern China (Guangdong and Hongkong), whereas three out of the four species in subclade 2 are endemics of south-western China (Yunnan).

**Figure 5.**
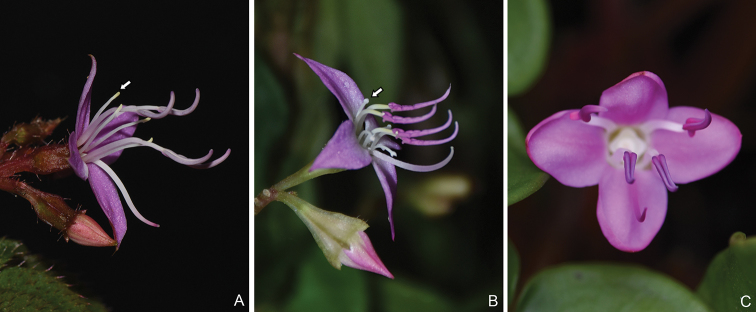
Stamen number and morphology of *Fordiophytonbreviscapum*, *Phyllagathiselattandra* and *P.tetrandra*. **A***F.breviscapum* from Ruyuan, Guangdong, China, 8 stamens with the shorter stamens greatly reduced **B***P.elattandra* from Guiping, Guangxi, China, 8 stamens with the shorter stamens sterile **C***P.tetrandra* from Xichou, Yunnan, China, 4 stamens. Arrows indicate anther of the shorter stamens.

The currently circumscribed *Fordiophyton* is not monophyletic, as *F.breviscapum* appears to be related to *Phyllagathistetrandra* and *P.elattandra*, rather than to other members of the same genus. *Fordiophytonbreviscapum* is morphologically most closely related to *F.degeneratum* (C. Chen) Y. F. Deng & T. L. Wu, which was not included in the phylogenetic analyses. These two species, as well as *P.tetrandra* and *P.elattandra*, have been treated in *Stapfiophyton* (Li 1944; Chen 1984a, b). Interestingly, these four species share some common features, such as hypanthium distinctly 4-sided and the inner whorl of stamens greatly reduced (*F.breviscapum*), sterile (*F.degeneratum* and *P.elattandra*) or undeveloped (*P.tetrandra*) (Fig. 5). As the relationships amongst these species are only weakly supported, their generic placement remains unclear, pending further study.

*Fordiophytondamingshanense* S. Y. Liu & X. Q. Ning is another species which was not sampled in previous and present phylogenetic studies. It highly resembles *F.faberi* in habit, leaf morphology and stamen morphology. Geographically, it occurs in Guangxi, where *F.faberi* also occurs. Morphology and distribution imply that *F.damingshanense* is probably a member of subclade 1.

### Phylogenetic position and specific status of *F.jinpingense*

The generic placement of *F.jinpingense* is supported by morphological and phylogenetic data. Its 4-merous flowers, eight distinctly dimorphic stamens and the connectives basally not calcarate fit perfectly well with the morphological circumscription of *Fordiophyton*. Phylogenetic analyses also showed that *F.jinpingense* was nested within the same clade, together with the type of *Fordiophyton*, *F.faberi*.

**Figure 6.**
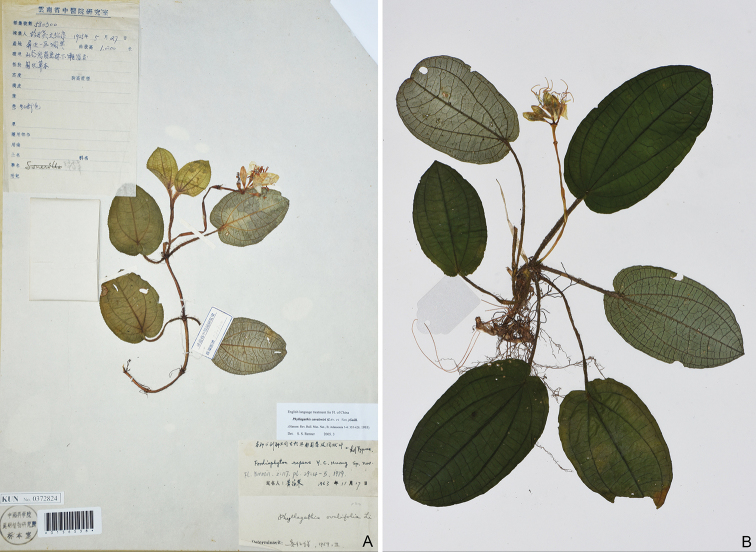
**A** Y. Y. Hu and S. K. Wen 580300 (KUN) collected from Pingbian County, Yunnan, China, holotype of *Fordiophytonrepens***B** Y. Liu 728 (A) collected from Ma-an-di, Jinping County, Yunnan, China, holotype of *Fordiophytonjinpingense*.

At the molecular level, pairwise sequence divergence at the nrITS region between *F.jinpingense* and other species of *Fordiophyton* ranges from eight to 42 nucleotide substitutions, which is equivalent to the number of substitutions between other species of *Fordiophyton* (ranging from 14 to 47 nucleotide substitutions). Molecular divergence, therefore, indicates that *F.jinpingense* is well diverged from other members of the genus. Morphologically, the basal rosette of leaves of *F.jinpingense* makes it quite distinct from species with erect, leafy stems, viz. *F.cordifolium*, *F.faberi*, *F.longipes* and *F.strictum*. It closely resembles *F.brevicaule*, *F.chenii*, *F.huizhouense*, *F.peperomiifolium* and *F.zhuangiae* in habit, but differs from *F.chenii* and *F.zhuangiae* in the unwinged, villous petioles (vs. winged and glabrous), from *F.huizhouense* and *F.peperomiifolium* in stems with distinct internodes (vs. indistinct) and from *F.brevicaule* in longer petioles (3–16 cm vs. 1–3 cm), larger leaf blades (6–16.5 × 4.5–13 cm vs. 3.5–8 × 2–5 cm) and umbellate inflorescence (vs. cymose paniculate). In fact, the phylogenetic analyses showed that *F.jinpingense* is most closely related to *F.repens* rather than to the above species. *Fordiophytonrepens* is narrowly endemic to Pingbian County, south-eastern Yunnan. The two are similar in having villous petiole and leaf blade, umbellate inflorescence and anther base of longer stamens forming a forked spur. Nevertheless, they differ markedly in stem morphology (short, obtusely 4-sided vs. long, 4-angular), habit (erect vs. creeping) (Fig. 6), leaf size (6–16.5 × 4.5–13 cm vs. 4–7.5 × 4–6.5 cm) and flower number per inflorescence (5–13 vs. 3–6). Therefore, both molecular and morphological evidence justify the recognition of *F.jinpingense* as a distinct species.

## Taxonomic treatment

### Fordiophyton jinpingense

Taxon classificationPlantaeMyrtalesMelastomataceae

J.H.Dai & Z.Y.Yu sp. nov.

urn:lsid:ipni.org:names:60478839-2

Figures 2 , 3

#### Type.

CHINA. Yunnan: Jinping County, Ma-an-di town, 900–1900 m alt., damp but well drained places in forest, 10 Mar 2019, Ying Liu 728 (holotype: A; isotype: SYS).

#### Diagnosis.

Differs from *F.repens* in having slightly obtusely 4-sided short stem (vs. 4-angular, long and creeping), mature leaves 6–16.5 × 4.5–13 cm (vs. 4–7.5 × 4–6.5 cm) sparsely and shallowly dentate leaf margin with each tooth having a caducous terminal seta (vs. densely denticulate, persistent) and inflorescence 5–13-flowered (vs. 3–6-flowered).

#### Description.

Perennial herbs, 10–14 cm tall (including inflorescence). Stems 2–5 cm long, slightly obtusely 4-sided, sometimes branched, villous with multiseriate hairs. Petiole 3–16 cm long, densely villous with multiseriate hairs; leaf blade ovate-oblong to ovate-orbicular, 6–16.5 × 4.5–13 cm, papery, adaxially green to dark green, abaxially pale green or sometimes purplish-red, villous with multiseriate hairs on veins, both surfaces inconspicuously pubescent with very short, appressed uniseriate hairs, secondary veins 3 or 4 on each side of midvein, base cordate, margin sparsely and shallowly dentate with each tooth having a terminal seta when young but caducous at maturity, apex short acute, obtuse or retuse. Inflorescences terminal and axillary, umbellate, 5–13-flowered; peduncle 9–14 cm long, sometimes white maculate, bearing several multiseriate hairs at nodes, otherwise glabrous; bracts caducous, oblong, 1–3 cm long, one pair (rarely two) in middle or lower part and another two pairs enclosing the flowers. Pedicels 4–10 mm long, glabrous. Hypanthium funnel-shaped, ca. 10 mm long, obtusely 4-sided, glabrous. Calyx lobes narrowly triangular-ovate, 2–5 × 1–2 mm, margin entire, apex obtuse or acute, caducous. Petals pink, obovate, ca. 16 × 8 mm, oblique. Longer stamens pink; filaments ca. 9 mm; anthers ca. 13 mm long, linear, curved, base lengthened into a forked, curved spur, connective bulging basally. Shorter stamens yellowish; filaments ca. 6 mm long; anthers oblong, 3–4 mm long, base obtusely forked, connective base slightly bulging. Ovary obovate, apex with a membranous ciliate, 4-lobed crown. Capsule funnelform-campanulate, ca. 6 mm in diam., apex 4-lobed, crown not exserted from calyx; hypanthium exceeding capsule, glabrous. Seeds numerous.

#### Phenology.

Flowering March–April, fruiting April–May.

#### Etymology.

The specific epithet is derived from Jinping County, the type locality of *Fordiophytonjinpingense*.

#### Distribution.

**Figure 7.**
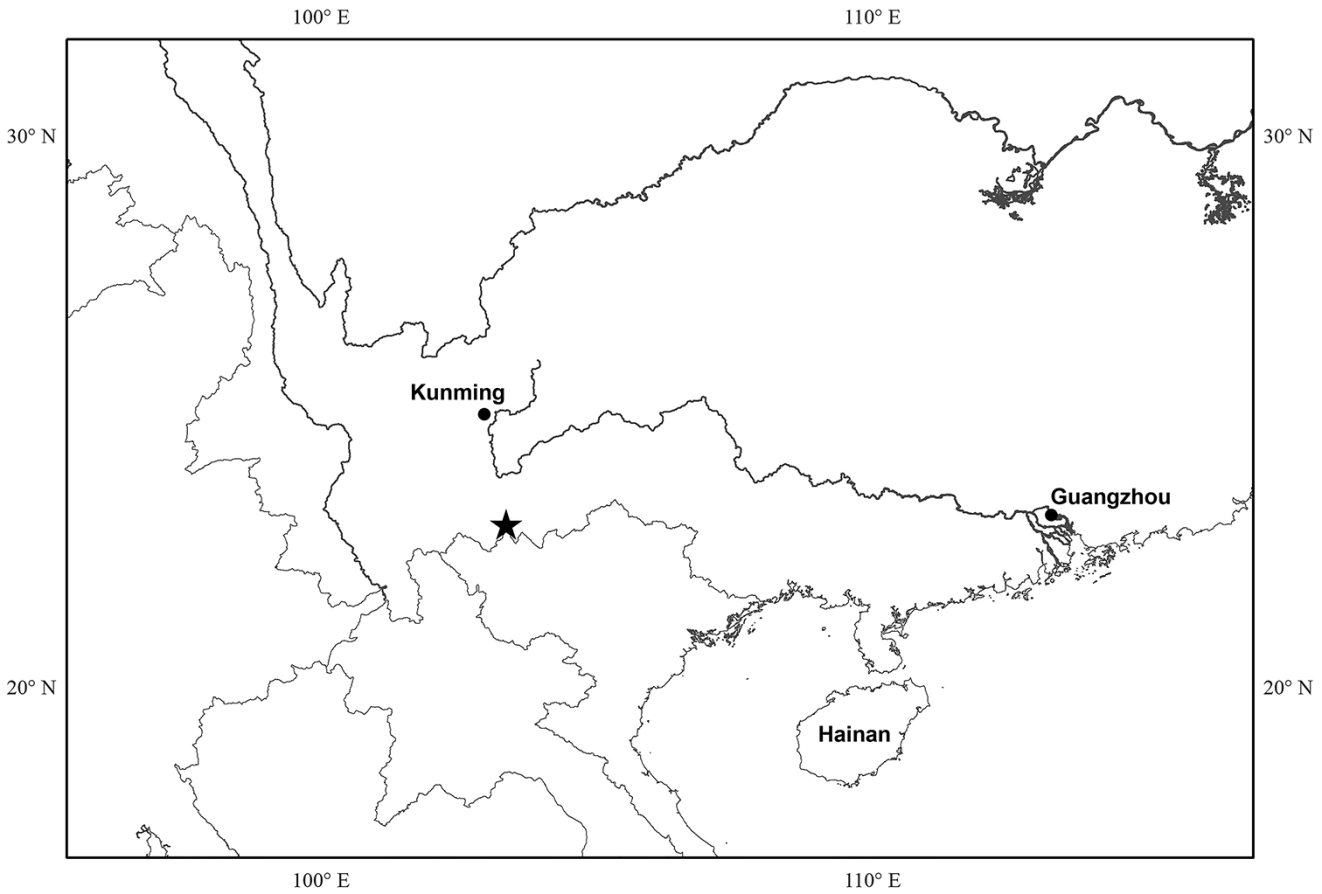
Distribution of *Fordiophytonjinpingense*.

*Fordiophytonjinpingense* is currently known only from Jinping County, south-eastern Yunnan, China (Fig. 7). It occurs in dense or open forests, often in damp, shaded, but well drained places, such as on steep slopes, at 900–1900 m alt.

### Key to the species of *Fordiophyton*

**Table:** 

1	Leaves in a basal or sub-basal rosette	**2**
–	Leaves cauline	**7**
2	Petiole winged and glabrous	**3**
–	Petiole unwinged, densely or sparsely hairy	**4**
3	Petioles 8–18 cm long; leaf blade 9–13 × 9–12 cm; secondary veins 4 on each side of midvein; hypanthium and calyx lobes hairy	*** F. chenii ***
–	Petioles 2–4 cm long; leaf blade 4–9 × 2–4 cm; secondary veins 2 or 3 on each side of midvein; hypanthium and calyx lobes glabrous	*** F. zhuangiae ***
4	Internodes of stems distinct	**5**
–	Internodes of stems indistinct	**6**
5	Internodes glabrous; petioles 1–3 cm long; leaf blade 3.5–8 × 2–5 cm; inflorescence cymose-paniculate; anthers of longer stamens forming an obtuse forked spur at base	*** F. brevicaule ***
–	Internodes hairy; petioles 3–16 cm long; leaf blade 6–16.5 × 4.5–13 cm; inflorescence umbellate; anthers of longer stamens forming a forked, curved spur at base	*** F. jinpingense ***
6	Hypanthium glabrous; calyx lobes lanceolate, 6 × 2 mm; base of connective of longer stamens prolonged	*** F. huizhouense ***
–	Hypanthium sparsely hairy; calyx lobes triangular, 1 × 2 mm; base of connective of longer stamens not prolonged	*** F. peperomiifolium ***
7	Stem creeping	*** F. repens ***
–	Stem erect or at least erect in upper part	**8**
8	Stem less than 20 cm long	**9**
–	Stem more than 30 cm long	**10**
9	Stem winged; secondary veins 1 on each side of midvein; inner 4 stamens fertile	*** F. breviscapum ***
–	Stem not winged; secondary veins 3 or 4 on each side of midvein; inner 4 stamens sterile	*** F. degeneratum ***
10	Leaves of a pair highly unequal and asymmetric; petioles often less than 1 cm long; bracts cordate, ca. 4 mm long	*** F. strictum ***
–	Leaves of a pair equal or slightly unequal; petioles more than 2 cm long; bracts more or less ovate, often more than 1 cm long	**11**
11	Leaf blade cordate to ovate-cordate, secondary veins 4 or 5 on each side of midvein	*** F. cordifolium ***
–	Leaf blade broadly lanceolate, oblong, ovate, oblong-lanceolate to elliptic; secondary veins 2 or 3, rarely 4 (*F.damingshanense*) on each side of midvein	**12**
12	Inflorescences umbellate, peduncle winged	*** F. longipes ***
–	Inflorescences cymose-paniculate, umbellate or a pleiochasium, peduncle not winged	**13**
13	Inflorescences cymose-paniculate, umbellate, 13–20 cm long	*** F. faberi ***
–	Inflorescences pleiochasia, ca. 10 cm long	*** F. damingshanense ***

## Supplementary Material

XML Treatment for Fordiophyton jinpingense

## Supplementary materials

Supplementary material 1 Table S1. Source of materials studied and GenBank accession numbers for nrITS Data type: molecular data Explanation note: Newly generated sequences are indicated in bold. This dataset is made available under the Open Database License (http://opendatacommons.org/licenses/odbl/1.0/). The Open Database License (ODbL) is a license agreement intended to allow users to freely share, modify, and use this Dataset while maintaining this same freedom for others, provided that the original source and author(s) are credited.Jin-Hong Dai, Qiu-Jie Zhou, Zhi-Yong Yu, Ren-Chao Zhou, Ying Liu

Supplementary material 2 Table S2. Summary statistics of sequences used for phylogenetic analyses Data type: phylogenetic analyses Explanation note: PIS, parsimony-informative sites. This dataset is made available under the Open Database License (http://opendatacommons.org/licenses/odbl/1.0/). The Open Database License (ODbL) is a license agreement intended to allow users to freely share, modify, and use this Dataset while maintaining this same freedom for others, provided that the original source and author(s) are credited.Jin-Hong Dai, Qiu-Jie Zhou, Zhi-Yong Yu, Ren-Chao Zhou, Ying Liu

Supplementary material 3 Figure S1. Maximum likelihood phylogenetic tree of Sonerileae based on nrITS sequences. Data type: Figure Explanation note: Numbers above branches are bootstrap values obtained from maximum likelihood analyses and those below branches are Bayesian posterior probabilities (right) and bootstrap values (left) resulting from Bayesian inference and maximum parsimony analyses, respectively. Species of *Fordiophyton* are indicated in bold; box denotes the new species; asterisk denotes a branch collapsed in Bayesian inference or maximum parsimony analyses. This dataset is made available under the Open Database License (http://opendatacommons.org/licenses/odbl/1.0/). The Open Database License (ODbL) is a license agreement intended to allow users to freely share, modify, and use this Dataset while maintaining this same freedom for others, provided that the original source and author(s) are credited.Jin-Hong Dai, Qiu-Jie Zhou, Zhi-Yong Yu, Ren-Chao Zhou, Ying Liu
